# N-Doped carbon quantum dot–based ratiometric fluorescent nanosensor platforms for detection of gastric cancer-associated *Helicobacter pylori* genes

**DOI:** 10.1007/s00604-025-07004-4

**Published:** 2025-02-12

**Authors:** Dilek Öztürk, Mahmut Durmuş

**Affiliations:** Department of Chemistry, Faculty of Science, Gebze Technical University, Gebze, 41400 Kocaeli Türkiye

**Keywords:** Carbon quantum dots, Gastric cancer, *Helicobacter pylori*, Fluorescence sensors

## Abstract

Carbon quantum dot (CQD)–based fluorescent nanosensor platforms were developed using gastric cancer-associated *Heliobacter pylori *(*H. pylori*) genes. N-doped CQDs were synthesized using two different organic acids (citric acid and malic acid) and ethylenediamine by the microwave method. The photophysical and photochemical properties of the synthesized CQDs were investigated by ultraviolet–visible, fluorescence, and Fourier-transform infrared spectra. The surface of the synthesized N-doped CQDs was conjugated with single-stranded DNA (ssDNA), which is specific for gastric cancer. Ethidium bromide, a selective dye, shows enhanced fluorescence intensity upon intercalating with DNA. In the blue-emissive CQD-ssDNA nanoprobe system, the fluorescence intensity was quenched by ethidium bromide due to Förster resonance energy transfer (FRET) processes. When complementary ssDNA was introduced, the ethidium bromide strongly intercalated with the newly formed double-stranded DNA, shifting to a red emission. Using this ratiometric system, the detection method was improved for gastric cancer–associated genes, achieving a limit of detection (LOD) of 0.098 µM, within a concentration range 1.30 to 11.49 µM. Spike and recovery tests were also conducted to evaluate the precision of the presented method in synthetic saliva solutions, with recoveries ranging from 93.06% to 101.85% The performance of the nanosensors was compared using two different synthesized CQDs.

**Graphical abstract:**

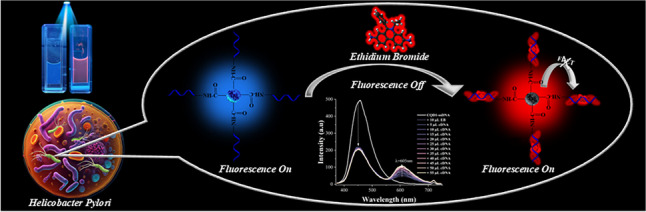

**Supplementary Information:**

The online version contains supplementary material available at 10.1007/s00604-025-07004-4.

## Introduction

*Helicobacter pylori* is a pathogen that causes various stomach disorders, such as reflux, indigestion, heartburn, and ulcers, and in advanced stages, it can lead to stomach cancer. *Helicobacter pylori* increases mucus permeability, penetrates the mucus membrane, and colonizes the deepest layer of the stomach epithelium. Furthermore, it neutralizes stomach acid by hydrolysing urea into NH₃ and CO₂ and damages the stomach mucosa by secreting enzymes such as mucinase, protease, and phospholipase [1].

*Helicobacter pylori* is classified as a Class 1 carcinogen by the World Health Organization (WHO). The sensitive and selective detection of *H. pylori*, which causes many diseases and reduces the quality of life in healthy individuals, is of great clinical importance for monitoring diseases associated with *H. pylori* infection [2]. The presence of *H. pylori* infection can be detected non-invasively by testing for antibodies through blood tests, detecting *H. pylori* antigen in stool, or using the urea breath test. However, more reliable methods for detecting *H. pylori* infection include taking a tissue sample from the stomach for rapid urease testing, histological examination, and microbial culture [3]. Traditional methods have some limitations because they require expensive equipment, expert people, and costly reagents [4]. Most importantly, all existing tests require clinical diagnosis and the use of a device. Therefore, alternative analytical biosensors are being developed for the simple, rapid, sensitive, and specific detection of bacteria. The greatest challenge in treating diseases associated with *H. pylori* infection is rapid diagnosis and treatment in the early stages.

Various methods based on different principles, such as optical, electrochemical, microfluidic, and paper-based approaches, have been developed for the rapid and point-of-care diagnosis of *H. pylori* infection [1]. In optical diagnosis methods, generally colorimetric and fluorometric detection methods are used for the diagnosis of the *H. pylori* pathogen. Colorimetric methods are based on colour change of pH indicators. Since the urease enzyme in the structure of *H. pylori* bacteria breaks down the urea in the environment into CO_2_ and NH_3_, it causes the pH of the environment to increase. Nevertheless, these methods give incorrect results in the presence of bacteria with similar properties (containing urease enzyme in its structure) [5]. The selectivity is important for determination of *H. pylori*, and this is provided by using specific DNA sequences, aptamers, antigens, antibodies, and DNAzymes [6–8]. In fluorometric detection methods, these biomarkers are used for selective and specific detection. Wu et al. reported a new aptamer-based fluorescent method which is specific for *H. pylori* whole cell, but their biosensor strategy requires high temperature (90 °C) to form aptamer complexes. This procedure has a significant drawback, which limits its practicality for point-of-care applications and increases the complexity of the detection process [9].

DNA-based biosensors stand out as a promising approach for the sensitive and selective detection of bacteria, offering high specificity through the recognition of target genetic sequences. According to the literature study on *H. pylori* detection using fluorometric method, Liu et al. introduced a new fluorescent DNA sensor with inorganic quantum dots (QDs) for detecting *H. pylori* DNA [10]. In another study, Shanehsaz et al. presented a fluorescence resonance energy transfer (FRET)–based method for detecting *H. pylori* DNA using two oligonucleotide probes labelled with CdTe QDs and 5-carboxytetramethylrhodamine (Tamra). Upon hybridization with the target DNA, Tamra emission occurs. The method demonstrated nanomolar-level detection of *H. pylori* nucleotide. However, the use of inorganic QDs poses certain drawbacks in these two studies. Inorganic QDs are composed of toxic heavy metals, raising environmental and biosafety concerns [11]. Carbon quantum dots (CQDs) are preferable materials due to their new class of fluorescence materials with adjustable emission wavelengths, high excitation efficiency, their ease of modification, lower environmental impact, and non-toxic nature [12]. Their functionalization flexibility and compatibility with ratiometric nanoquencher systems make them widely used in sensing applications [13–15]. For instance, Bu et al. utilized blue-emissive CQDs in a ratiometric fluorescence sensor for the detection of arginine and acetaminophen, leveraging DNA-templated copper nanoclusters [16]. Similarly, He et al. functionalized CQDs with aminophenylboronic acid via carbodiimide chemistry, creating a nanoquencher sensitive to dopamine and developing a ratiometric fluorescence sensor [17]. There are many DNA-based biosensor studies using CQDs in the literature [18–20], but there is no study for the detection of genes associated with *H. pylori*. This study was inspired by the approach utilized by Huang et al., who employed double-stranded DNA directly as the analyte.

**Scheme 1:**
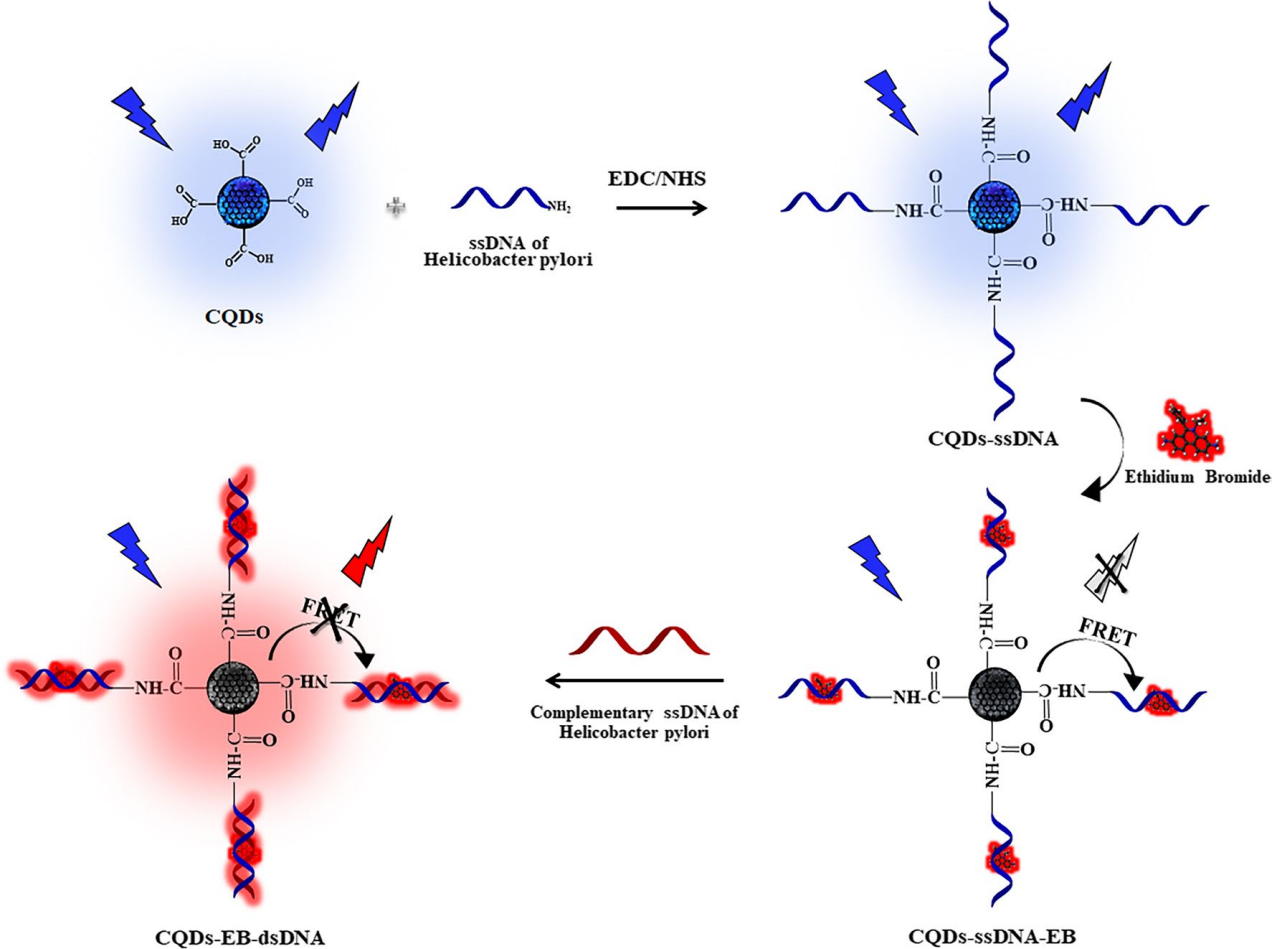
Developed biosensor mechanism for *H. pylori* detection

In this work, a sensor system was developed by generating double-stranded DNA within the biosensor environment. Specifically, two different N-doped carbon CQDs were synthesized, and their surfaces were modified with amino-functionalized single-stranded DNA (ssDNA) specific to *H. pylori* bacteria. The developed sensor had two stages; firstly, the fluorescence emissions of CQDs-ssDNA emitting different wavelengths were quenched by the electron transfer processes by ethidium bromide (EB) added to the medium. Then, when complementary ssDNA was added to the system, the red-emitting ethidium bromide interacted strongly with the hybridized double-stranded DNA through intercalation and gave fluorescence again. Thus, while the red fluorescence emission intensity of ethidium bromide increased, a proportional nanosensor system was developed by decreasing the blue fluorescence emission of CQDs. The developed sensing mechanism is given in Scheme 1.

## Materials and methods

Citric acid and malic acid were supplied by Carlo Erba; ethylenediamine, quinine sulphate, sodium hydrogen phosphate di-sodium hydrogen phosphate, sodium chloride, potassium chloride, calcium chloride, glucose, galactose, ascorbic acid, L-tryosine, thymine, L-cysteine, guanine, adenine, L-arginine, and BSA (bovine serum albumin), and phenolphthalein were supplied by Merck; sodium hydroxide, hydrochloric acid (37%), N-(3-dimethylaminopropyl)-N′-ethylcarbodiimide hydrochloride (EDC HCI), N-hydroxysuccinimide (NHS), and ethidium bromide were supplied by Sigma-Aldrich; methanol, ethanol, and acetone were supplied by Isolab. A microwave heater (Altus) and an ultrasonic bath (United ultrasonic cleaner) were used in the synthesis stages of N-doped CQDs.

Primer DNA strands were supplied by Sentebiolab Biotechnology Company. The primary DNA strands sent in lyophilized form were prepared in phosphate-buffered saline (PBS) solution with pH 7.4 and stored in portions ready for use at − 18 °C.

Probe DNA (ssDNA): 5′-NH_2_-(CH_2_)_6_–GCG TTC CAA AGG GCA GGA TCA TTG A-3′

Complementary DNA (cDNA): 5′-TCA ATG ATC CTG CCC TTT GGA ACG C-3′

Non-complementary DNA (non-cDNA): 5′-GAC CGT CGA AGT AAA GGG TTC CAT A-3′

### Synthesis of N-doped CQDs

**Fig. 1:**
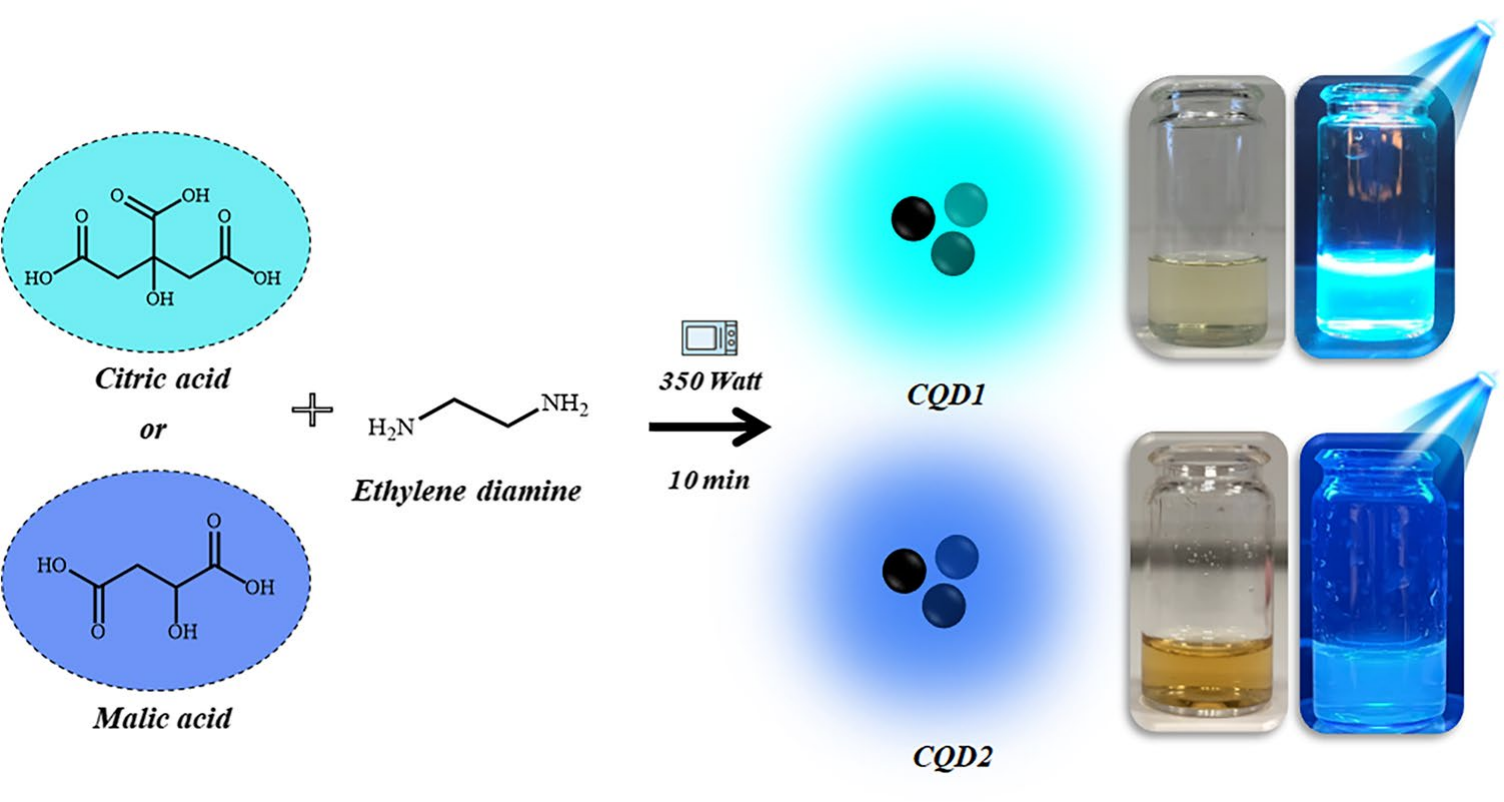
Synthesis scheme of N-doped CQDs and their images under daylight and UV light

The details of the N-doped CQDs synthesis, using the modified method applied by Ghirardello and his colleagues, are provided in the electronic supporting material [21]. The synthesis scheme of the N-doped CQDs and their images under daylight and ultraviolet (UV) light are given in Fig. 1. The physical and chemical structures of CQDs were examined by different methods such as transmission electron microscopy (TEM), X-ray diffraction (XRD), and Fourier-transform infrared (FTIR).

### Fluorescence quantum yield

Fluorescence quantum yield is an important parameter in the study of fluorescent materials because it gives insight into the efficiency of their luminescent properties. It is a measure of the conversion of absorbed light into emitted fluorescence by a molecule or material [22]. The fluorescence quantum yields (Φ_F_) of the synthesized N-doped CQDs were calculated using the following equation by a comparative method;

1 $$\Phi_{\mathrm F}={\mathrm\Phi}_{\mathrm F}\left(\mathrm{Std}\right)\frac{\mathrm F.\;A_{\mathrm{Std}}}{F_{\mathrm{Std}}.\;\mathrm A}$$

Terms *F* and *F*_Std_ represent the areas under the fluorescence emission curves for the N-doped CQDs and the standard reference material, respectively. Similarly, *A* and *A*_Std_ denote the absorbance values of the N-doped CQDs and the standard reference material at their respective excitation wavelengths. Quinine sulphate was used as a reference material and, its fluorescence quantum yields (Φ_*F*std_) is 0.54 in a 0.1-M H_2_SO_4_ solution [23].

### Amide coupling reaction for CQDs-ssDNA hybrid

The surface of the N-doped CQDs was functionalized with ssDNA using carbodiimide chemistry. The -COOH groups on the surface of N-doped CQDs were covalently linked to the -NH_2_ modified ssDNA through the EDC/NHS coupling method. 1 mg/mL 1 mL N-doped CQDs were prepared in a 0.01-M PBS pH:7.4 solution. 20 mg EDC and 10 mg NHS were added in this solution and stirred at 37 °C for 2 h for the activation of -COOH groups. A 20-μL activated CQD1 solution was mixed with 50 μL ssDNA (100 μM) for 2 h at 37 °C for conjugation [18]. Afterward, the total volume was adjusted to 370 μL using a 0.01-M PBS solution, and fluorescence signals were recorded. Similarly, a CQD2 solution (5 mg/mL in 1 mL) was activated following the same procedure. Then, 100 μL of the activated CQD2 solution was mixed with 50 μL ssDNA (100 μM) and incubated under identical conditions for conjugation.

## Results and discussion

### Characterization of N-doped CQDs

**Fig. 2:**
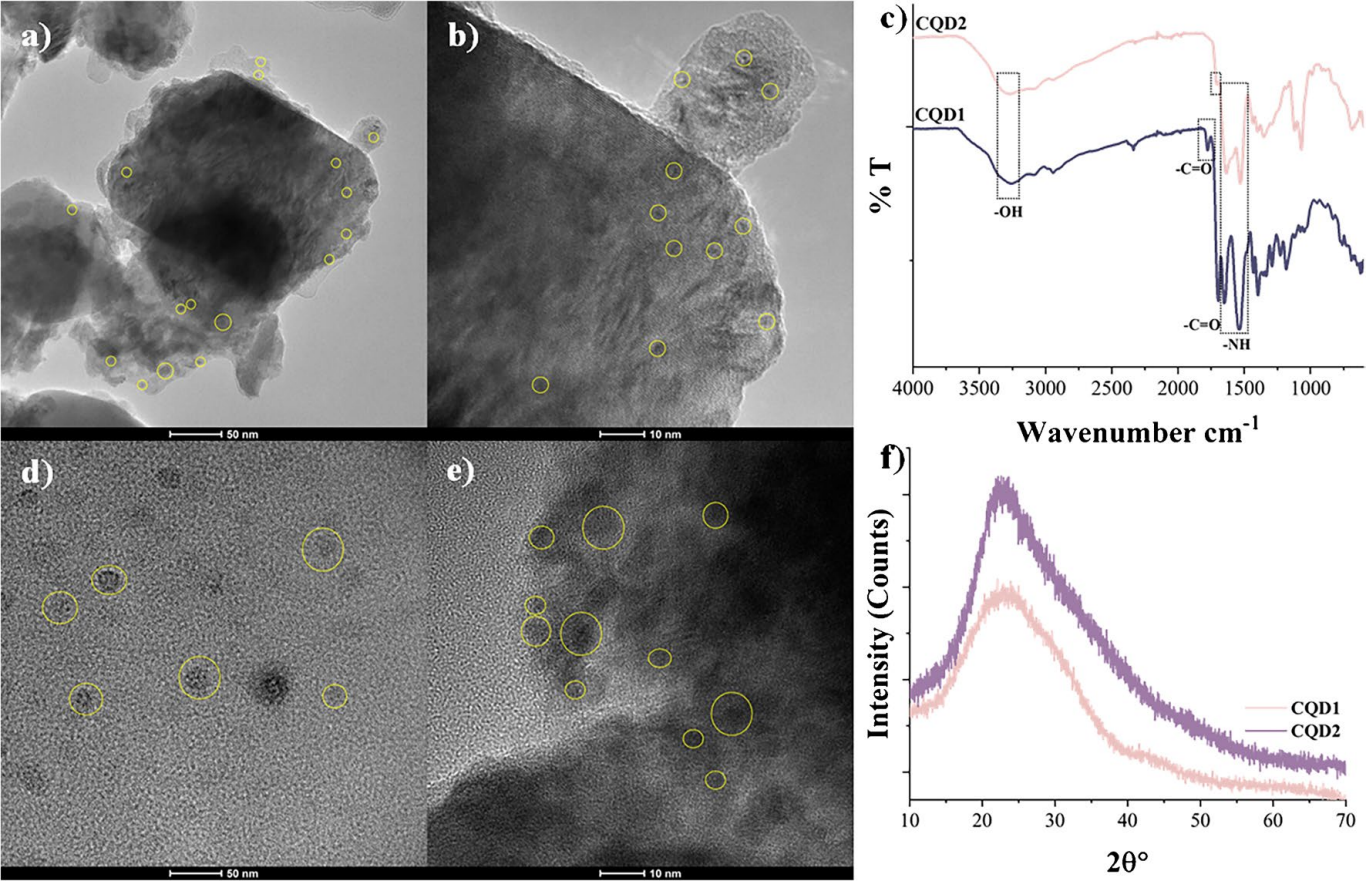
TEM images of synthesized N-doped CQDs: **a**, **b** CQD1, **d**, **e** CQD2; FTIR spectra (**c**) and XRD patterns (**f**) of synthesized N-doped CQDs

TEM images of N-doped CQDs are presented in Fig. 2. As seen in the figure, the sizes of materials were clearly nano-sized particles. However, obtaining high-resolution TEM images was challenging due to the high electron density of the N-doped CQDs and the low contrast between the N-doped CQDs and the carbon-coated TEM grids. Despite this, several particles were randomly selected and marked with circles in the figure, confirming the successful synthesis of nano-sized materials.

FTIR analysis was performed to obtain information about the chemical structure and functional groups of N-doped CQDs. According to the FTIR spectrum shown in Fig. 2c, the broad band at around 3262 cm^−1^ was attributed to the -OH group; the peak at 3083 cm^−1^ was attributed to the -NH functional group. There were peaks belonging to -CH stretching vibrations at 2945 cm^−1^, asymmetric stretching vibrations of -C-NH-C at 1177 cm^−1^, amide I band at 1653 cm^−1^ (-C = O stretching), amide II band belonging to -NH bending vibrations at 1536 cm^−1^, and -C = O vibration absorption band at 1780 and 1694 cm^−1^ [24].

The XRD patterns of synthesized of N-doped CQDs are given in Fig. 2f. Broad peaks at around 22 ϴ° indicate the 002 graphite lattice plane, consistent with the TEM images. These results indicate low graphitic carbon structure in materials [25].

### Photophysical properties of N-doped CQDs

N-doped CQDs exhibit fascinating photophysical properties, making them valuable for a wide range of applications such as bioimaging, sensing, and optoelectronics [26]. The doping of nitrogen into the carbon structure introduces unique electronic characteristics, enhancing their quantum yield, photoluminescence, and stability [27]. These properties are influenced by factors such as size, surface functionality, and the presence of nitrogen-containing functional groups, which can modulate the emission wavelength and intensity of the N-doped CQDs. By controlling the synthesis parameters, N-doped CQDs can be tailored for specific photophysical behaviours, including high fluorescence quantum efficiency, tuneable emission, and strong photostability under various environmental conditions [28].

**Fig. 3:**
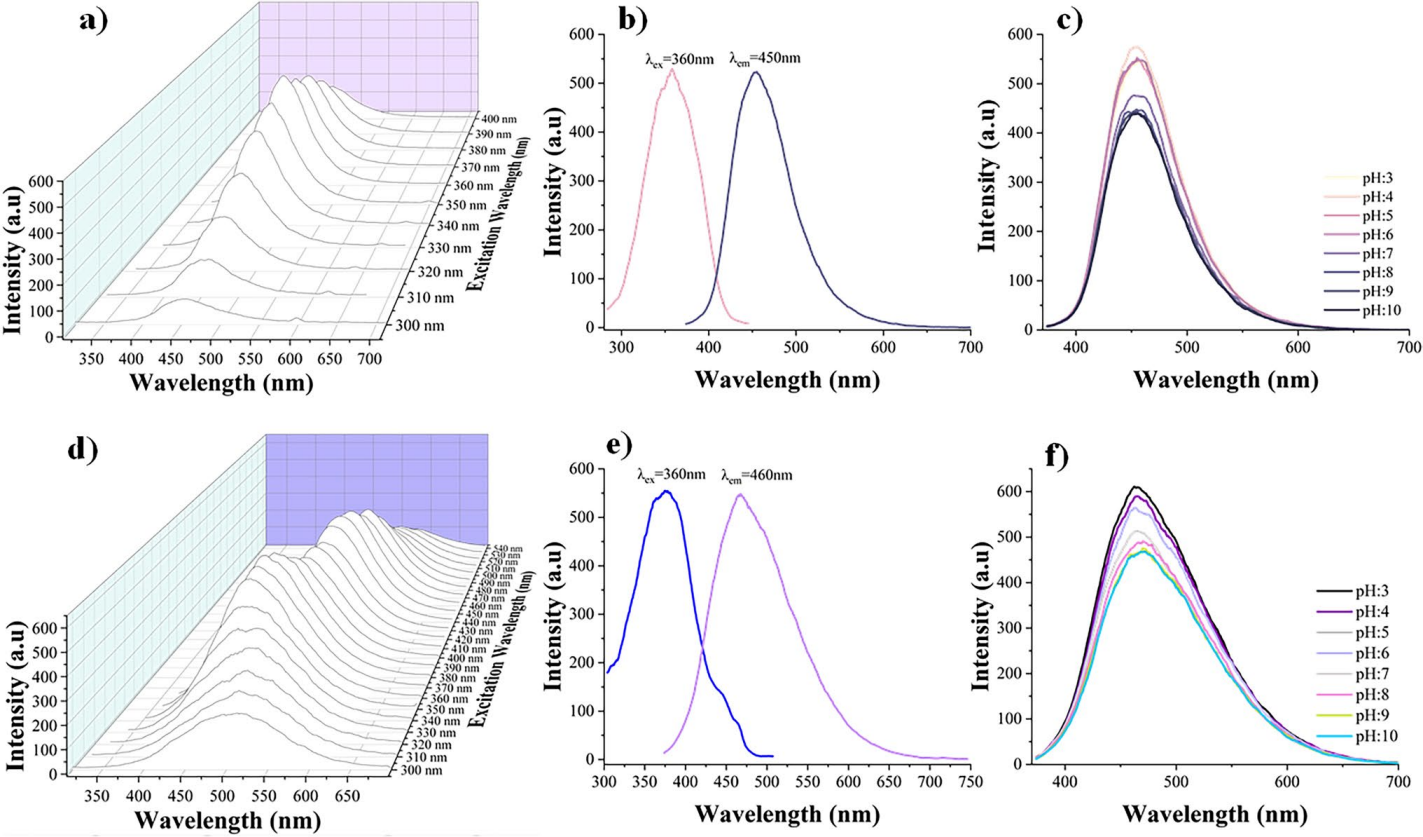
Fluorescence spectra of CQD1 (**a**–**c**) and CQD2 (**d**–**f**): **a**, **d** different excitation wavelengths; **b**, **e** excitation–emission graphs; **c**, **f** pH effects on fluorescence intensity (C_CQD1_: 62 ppm, C_CQD2_: 1667 ppm)

The fluorescence spectra and effect of pH on fluorescence properties of N-doped CQDs are given in Fig. 3. The highest emission wavelength of CQD1 solution was determined by exciting in the range of 300 to 400 nm, increasing by 10 nm increments (Fig. 3a). It was found that when excited at 360 nm, it had the highest emission at 450 nm (Fig. 3b). The effect of different pH values on the emission intensity of CQD1 had also been examined, and it was revealed that acidic media (pH below 5) increased the fluorescence intensity, while neutral and basic media did not cause any change in fluorescence intensity (Fig. 3c).

The CQD2 exhibited increasing emission intensity in an aqueous solution when excited within the 300–400-nm range, increasing by 10 nm increments (Fig. 3d). Although the fluorescence intensity decreased beyond 400 nm, a significant and readable signal was still observed between 400 and 500 nm. The emission in this wavelength range can be utilized for various analyses. The maximum emission intensity was achieved at 460 nm under excitation at 360 nm (Fig. 3e). The effect of different pH values on the fluorescence emission intensity of CQD2 was also examined. In Fig. 3f, it was observed that the fluorescence emission of CQD2 decreased by the changing of pH level from 3 to 10. The concentrations of CQDs varied significantly to achieve suitable fluorescence intensity. The highest fluorescence intensity was observed at the concentration of 62 ppm for CQD1 and 1667 ppm for CQD2 in aqueous solutions. The physical and photophysical properties of the N-doped CQDs used for the developed sensor are presented in Table S1. CQDs generally exhibit specifically broad absorption peaks between 250 and 350 nm in the UV region. CQD1 exhibited two absorption peaks at 240 and 350 nm. While near the peaks around 240 nm were attributed to π–π* transitions, which arise from the C = C bondsin the carbon core, the peaks around 340 nm corresponded to *n*–π* transitions from C = O bonds as surface functional groups [29]. CQD2 also exhibited two absorption peaks at 340 and 450 nm, which could be attributed to transitions from *σ* and *π* orbitals the highest occupied molecular orbitals (HOMO) to the lowest unoccupied molecular orbitals (LUMO). The presence of carbonyl and amino functional groups induces red shifts in the absorption band of the UV–Vis spectra, which results from alterations in the HOMO–LUMO energy levels due to surface functionalization [29].

In the scope of the functionalization of nanomaterials, determining the functional groups on the surface, particularly the amount of -COOH groups, is crucial for improving the properties of the materials. The amount of -COOH groups was determined using the acid–base titration method; CQD1 was found to have 2.025 ± 0.075 μmol of -COOH groups, and CQD2 was found to have 0.885 ± 0.05 μmol of -COOH groups. The amount of the -COOH groups are related to pH of the CQDs. CQD1 exhibited more acidic properties than CQD2. The fluorescence quantum yield of CQD1 was found to be 23.0%, while the fluorescence quantum yield of CQD2 was found to be 15.3%. The reason for the higher concentration of CQD2 in the emission measurements was its lower fluorescence quantum yield compared to CQD1 (Table S1).

The zeta potential and functional groups on the surface of nanoparticles are closely related to each other. Specifically, negatively charged groups, such as carboxylic acid moieties, contribute to a decrease in zeta potential, while positively charged groups, such as quaternized amino functional groups, are associated with an increase in zeta potential values [30]. The zeta potential value of CQD1 was found more negative than CQD2. These results were consistent across different pH levels and were related to the amount of -COOH groups (Table S1).

### Biosensor detection strategies, optimized parameters, and performances

Ethidium bromide is a dye that intercalates into the structure of double-stranded DNA. It is a phenanthridine monomer dye that increases its fluorescence emission 20 to 25 times when intercalated into double-stranded DNA [31]. The dye inserts between adjacent base pairs of the double-stranded DNA molecule [32], and the observed increase in fluorescence upon ultraviolet light excitation of ethidium bromide is thought to be partially due to energy transfer from the DNA bases to the dye [33]. Ethidium bromide emits fluorescence much more brightly in the presence of double-stranded DNA compared to ssDNA [34].

In a study conducted by Huang et al. in 2015, a ratiometric sensor system was developed for the detection of double-stranded DNA using CQDs and ethidium bromide [35]. In this study, based on this principle, ssDNA conjugated to CQDs had low fluorescence intensity in the presence of ethidium bromide. However, when complementary single-stranded DNA (cDNA) was introduced, hybridization occurred, forming a double-stranded structure, which resulted in increased fluorescence emission of ethidium bromide. As double-stranded DNA was formed in the presence of both CQDs and ethidium bromide, the fluorescence emission intensity of ethidium bromide around 605 nm proportionally increased when excited in the excitation region of both CQDs.

**Fig. 4:**
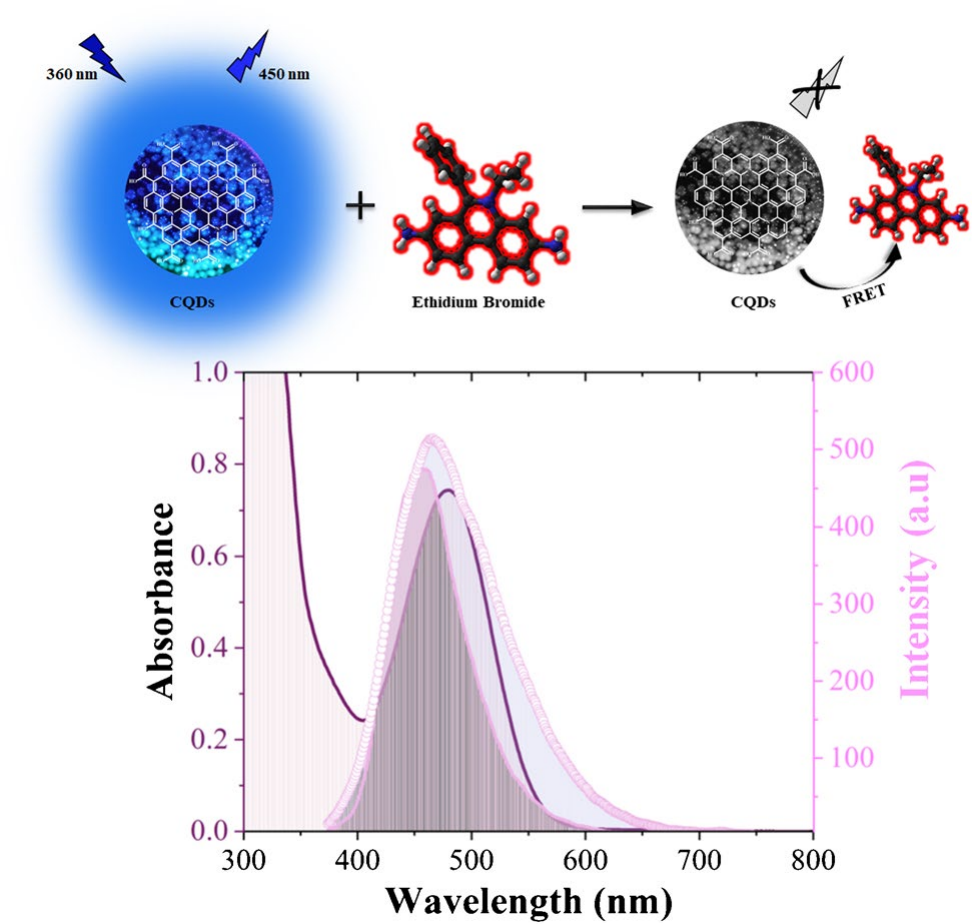
FRET mechanism and overlap graph between absorbance spectrum of ethidium bromide and emission spectra of CQD1 and CQD2

The developed fluorescence biosensor operates based on the FRET mechanism, attributed to the overlap between the absorbance spectrum of ethidium bromide and the emission spectrum of the synthesized CQDs. The schematic representation of the FRET mechanism, along with the overlay of the spectral graphs, is provided in Fig. 4.

**Fig. 5:**
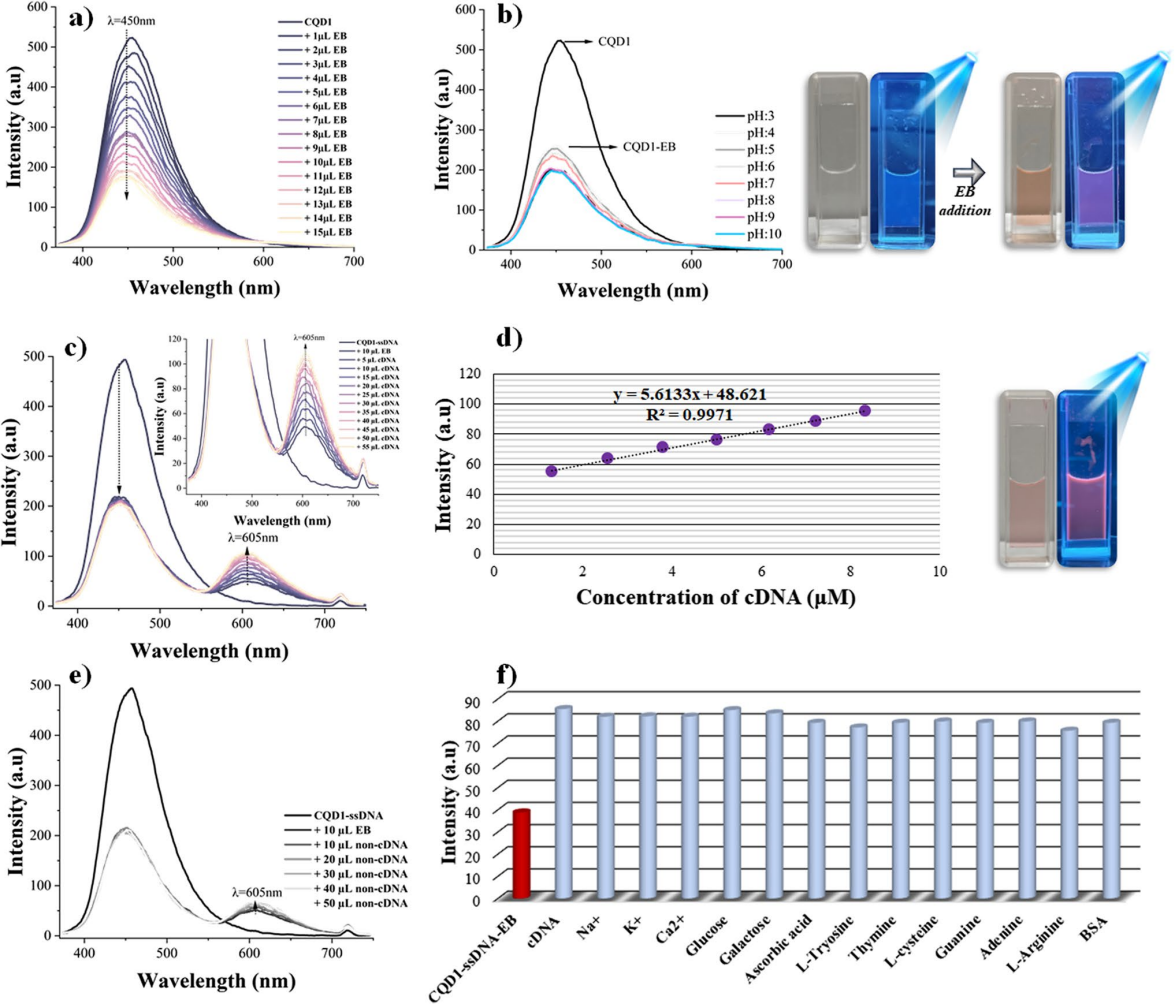
Fluorescence responses of the biosensor system: **a** intensity changes of CQD1 upon addition of ethidium bromide, **b** pH effect on the CQD1-EB quenched sensor system, **c** fluorescence ‘turn-on’ response upon addition of complementary DNA (100 μM), **d** linear relationship of fluorescence ‘turn-on’ efficiency, **e** selectivity study with non-complementary DNA (100 μM), and** f** interference study of the biosensor system

The CQD1 solution (62 ppm) and ethidium bromide solution (8.5 mM) were prepared in a 0.01-M PBS buffer at pH 7.4. The CQD1 solution, which exhibited the highest emission at 450 nm, was mixed with increasing concentrations of ethidium bromide solution, and the resulting fluorescence intensity was recorded, leading to fluorescence quenching (Fig. 5a). The decrease in the fluorescence intensity of CQD1 by the addition of ethidium bromide and the changes in fluorescence intensity of the CQD1-EB system at different pH values are presented in Fig. 5b. As seen in this figure, when the pH was varied between 3 and 10, no significant change in fluorescence intensity was observed. Considering its suitability for biological environments, pH 7 was determined to be the most appropriate value, and further studies were conducted at this pH. It was also observed that CQD1 exhibited blue emission under UV light, and when this solution was mixed with ethidium bromide, the colour of the solution turned to orange, giving off a low orange-red emission.

In accordance with the developed biosensor mechanism, the fluorescence intensity of the CQD1-ssDNA solution was initially recorded. Subsequently, an optimized amount of ethidium bromide solution was introduced to quench the fluorescence emission at 450 nm. After the quenched CQD1-ssDNA-EB sensor system was obtained, 10 µL of 100 µM complementary DNA (cDNA) was added to this biosensor system to recover the fluorescence emission at 605 nm. Figures 5c–d illustrate the recovery of fluorescence emission and the linear relationship between the recovered fluorescence intensity and the complementary DNA (cDNA) concentration (*R*^2^ = 0.9971). In addition, the image of the solution under UV light, which emitted red–orange fluorescence due to energy transfer from ethidium bromide binding to the hybridized DNA strands in the medium, is also presented in Fig. 5.

The selectivity study was conducted using 100 µM non-complementary DNA (non-cDNA). The effect on fluorescence intensity of increasing amounts of non-complementary DNA (non-cDNA) is given in Fig. 5e. The emission peak at 605 nm did not significantly increase as seen from the spectra. The interference study was also performed by the addition of 0.1 M 10 μL Na^+^, K^+^ Ca^2+^, glucose, galactose, ascorbic acid, L-tryosine, thymine, L-cysteine, guanine, adenine, L-arginine, and BSA (bovine serum albumin) solutions to biosensor environment. As shown in Fig. 5f, these interference materials did not significantly affect to the sensing performance. Fluorescence spectra of this interference study are given in Figure S1.

**Fig. 6:**
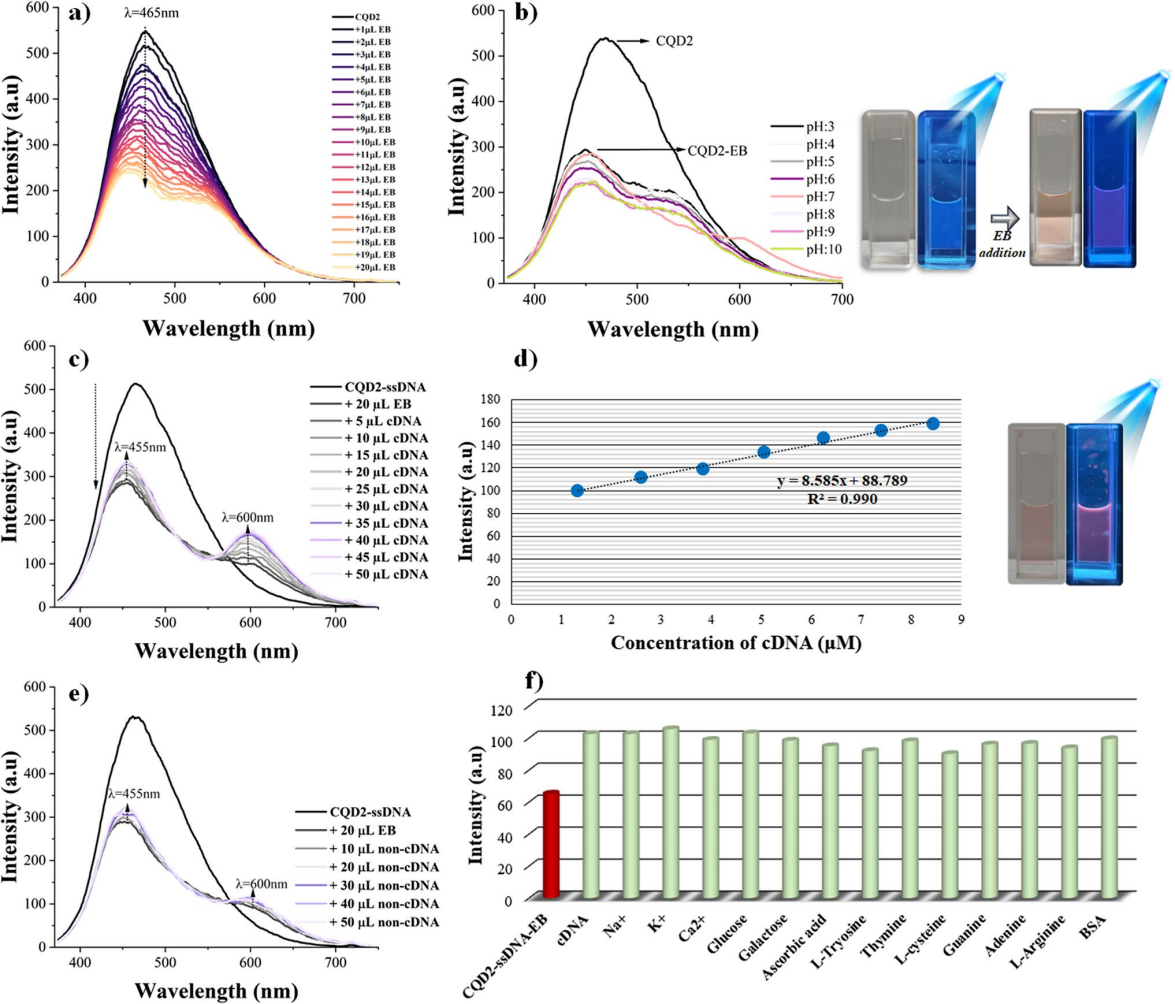
Fluorescence responses of the biosensor system: **a** intensity changes of CQD2 upon addition of ethidium bromide, **b** pH effect on the CQD2-EB quenched sensor system, **c** fluorescence ‘turn-on’ response upon addition of complementary DNA (100 μM), **d** linear relationship of fluorescence ‘turn-on’ efficiency,** e** selectivity study with non-complementary DNA (100 μM), and **f** interference study of the biosensor system

Similar biosensor studies were conducted for CQD2, and results are given in Fig. 6. The CQD2 solution (1667 ppm) and ethidium bromide solution (2.5 mM) were prepared in a 0.01-M PBS buffer at pH 7.4. The CQD2 solution, which exhibited the highest emission at 465 nm, was mixed with increasing concentrations of ethidium bromide solution, and the resulting fluorescence intensity was recorded, leading to fluorescence quenching. As ethidium bromide was added, a shoulder-shaped new peak at around 530 nm was also observed. The decrease in the fluorescence intensity of CQD2 by the addition of ethidium bromide and the changes in fluorescence intensity of the CQD2-EB system at different pH values are given in Fig. 6b. As seen in this figure, fluorescence intensity increased in acidic conditions while it decreased in basic conditions. Considering suitability for biological environments, pH 7 was determined to be the most appropriate value, and further studies were conducted at this pH.

After obtaining the quenched CQD2-ssDNA-EB sensor system, 10 µL of 100 µM complementary DNA (cDNA) was added to this sensor system, resulting in the recovery of fluorescence at both 455 nm and 600 nm (Fig. 6c). As increasing concentrations of complementary cDNA were added, a rise in fluorescence intensity was recorded. As seen in Fig. 6d, a linear relationship was observed between the concentration of complementary cDNA and the fluorescence emission intensity (*R*^2^ = 0.990). Also, selectivity studies were conducted with non-complementary DNA (non-cDNA); the fluorescence intensity recovery did not obtain significantly (Fig. 6e). Similarly, an interference study was conducted for CQD2. As shown in Fig. 6f, these interfering substances did not significantly affect the biosensor’s detection system. The corresponding fluorescence spectra are provided in Figure S2.

In Table 1, the LOD, limit of quantification (LOQ) values, and linear range limits of the developed sensors are presented. When CQD1 was used, genes associated with *H. pylori* could be detected with a LOD value of 0.098 µM. In comparison, the LOD value was 0.28 µM when CQD2 was used. The LOD value was calculated according to the rule of three times the standard deviation (LOD = 3Std/S), and the LOQ value was calculated according to the rule of 10 times the standard deviation (LOQ = 10Std/S). This study presents a novel approach by utilizing ethidium bromide for the specific detection of a target gene. The precision of the developed biosensor was assessed at three different cDNA concentrations using synthetic saliva solutions [36]. As presented in Table 1, the recovery ranged from 93.06% to 101.85%, while the relative standard deviation (RSD%), a key parameter of precision, was between 0.09% and 3.78%, well below the 5% threshold. These findings confirm the method's acceptability, repeatability, and reliability.

**Table 1:** Analytical parameters of developed CQDs-based biosensors

CQDs	LOD (μM)	LOQ (μM)	Linear range (μM)	Added (μM)	Found (μM)	% Recovery	% RSD
CQD1	0.098	0.30	1.30–11.49	2.56	2.55	97.05 ± 3.75	3.78
				3.80	3.77	99.15 ± 0.03	0.82
				4.26	4.08	95.78 ± 2.74	2.87
CQD2	0.28	0.92	1.33–11.76	2.59	2.64	101.85 ± 3.29	3.23
				3.85	3.73	97.14 ± 0.08	0.09
				4.30	4.00	93.06 ± 1.58	1.69

Photostability and interaction time between the analyte and biosensor material are crucial for developed analytical methods. In Figure S3, photostability and interaction time results are given for each biosensing system. The graph shows the relationship between interaction time and fluorescence intensity for varying concentrations of complementary DNA (cDNA) when using CQD1 and CQD2 as the biosensor material. As seen in Figures S3a and S3b, measurements were recorded at 10-s intervals for the maximum fluorescence signal change, during which no significant variation in fluorescence intensity was observed. Notably, the fluorescence signals stabilized within the first 10 s for both biosensors, indicating rapid equilibrium in their interaction with the analyte. In photostability study, as illustrated in Figures S3c and S3d, the fluorescence signal remained stable over a 30-min period, demonstrating the high photostability of the developed biosensors.

Optical DNA biosensors in the literature typically detect double-stranded DNA [37]. Bai et al. used methylene blue for DNA detection, achieving a detection limit of 1 μM for calf thymus DNA [38]. Similarly, Huang et al. reported a detection limit of 0.47 μM for calf thymus DNA using ethidium bromide with double-stranded DNA directly [35]. Although the studies by Bai et al. and Huang et al. employ optical DNA biosensors for detecting double-stranded DNA, their selectivity is limited. Similar studies are given in Table S2, these biosensors can respond to any double-stranded DNA present in the environment, lacking specificity. In contrast, the current study offers a significant improvement, demonstrating both a lower limit of detection (LOD) of 0.098 μM and enhanced selectivity compared to the literature.

### Biosensor responses in 3D fluorescence graphs and lifetimes

**Fig. 7:**
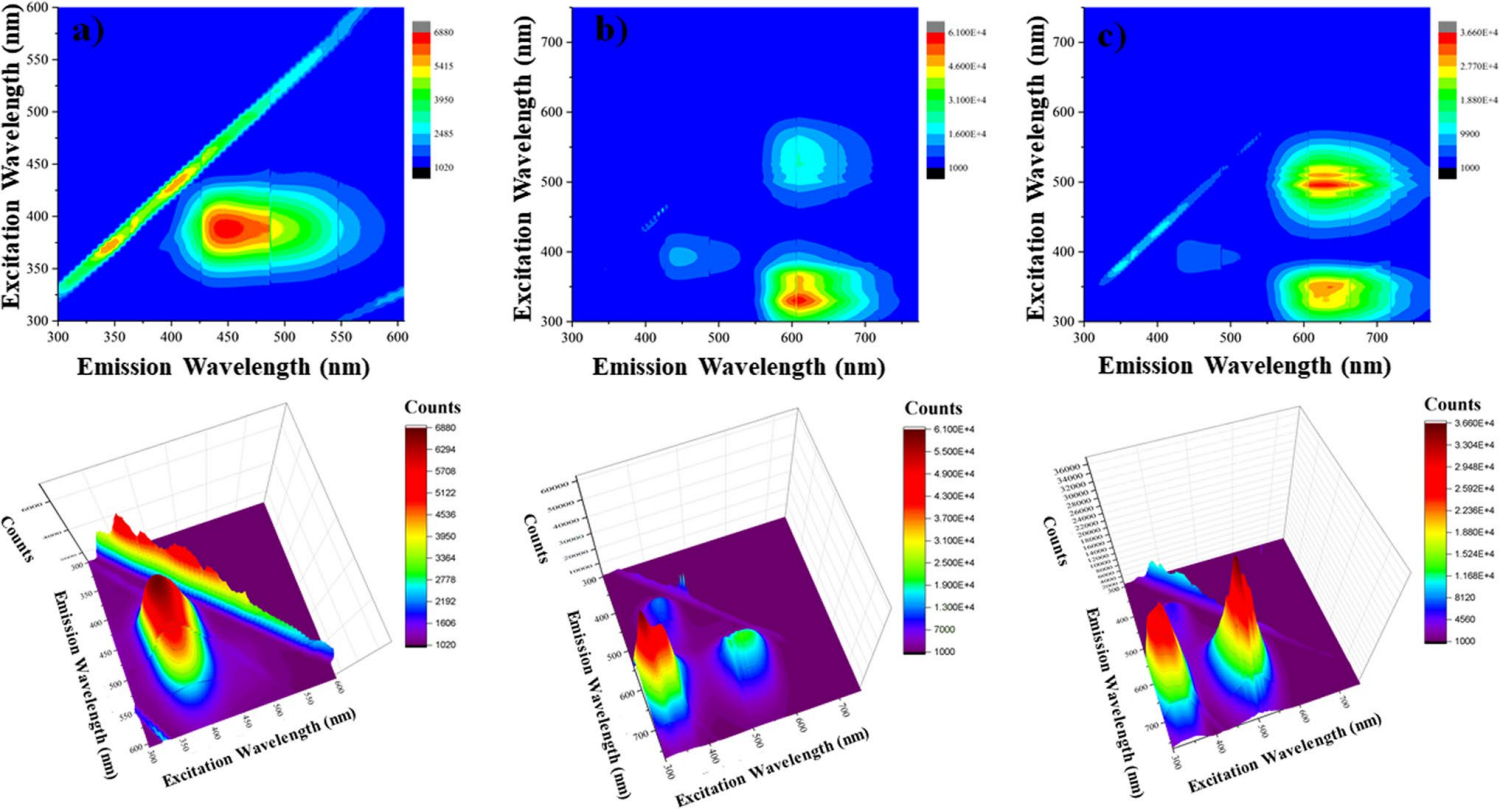
3D fluorescence counter plots of **a** CQD1-ssDNA, **b** CQD1-ssDNA + EB, and **c** CQD1-ssDNA + EB + dsDNA

The 3D emission graphs of the developed sensor system are presented in Fig. 7. It has been confirmed three-dimensionally that the fluorescence emission of CQD1-ssDNA at 450 nm was quenched by the addition of ethidium bromide, and the emission intensity increased again at 605 nm by the addition of complementary DNA.

**Fig. 8:**
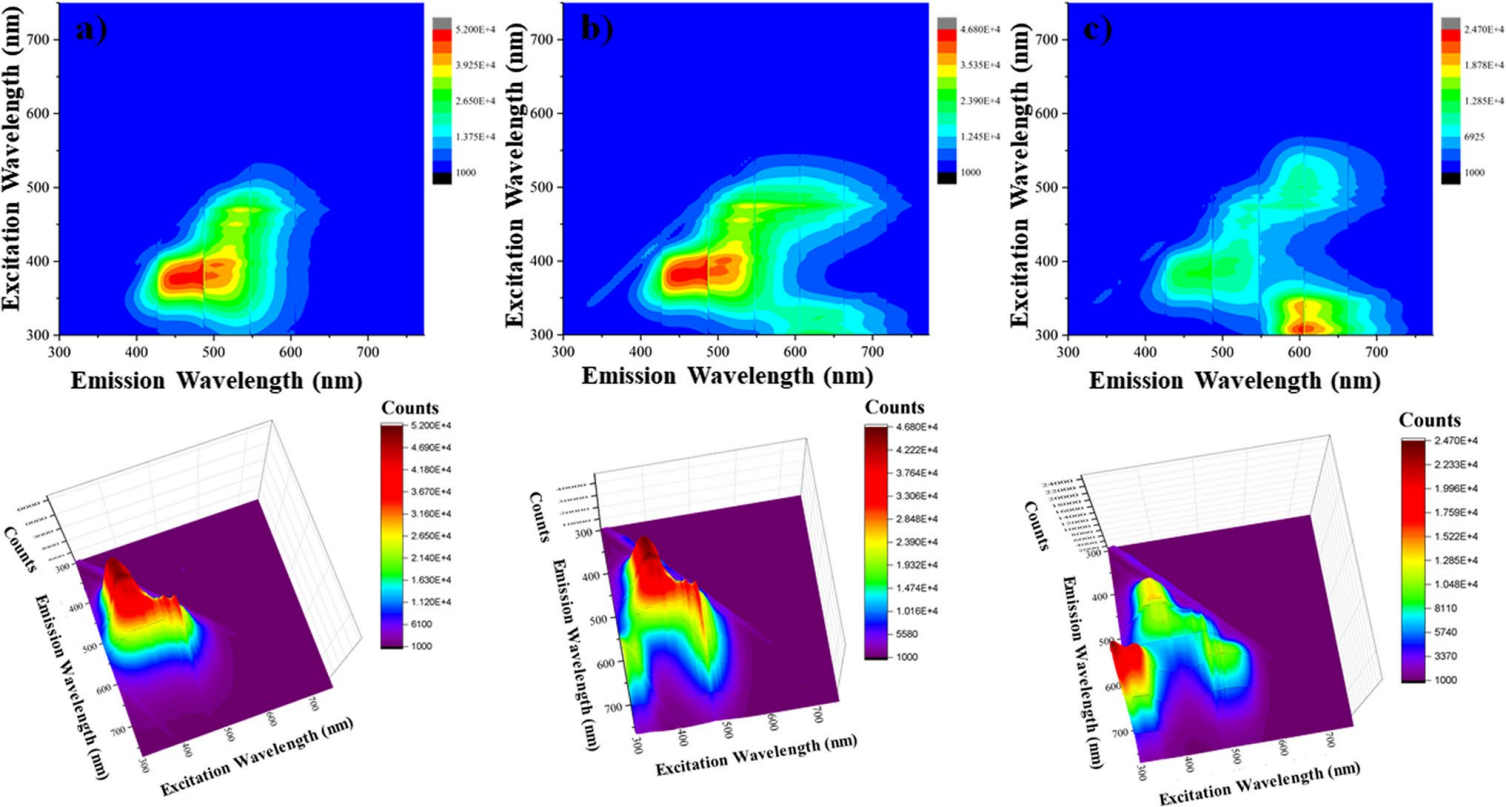
3D fluorescence counter plots of **a** CQD2-ssDNA, **b** CQD2-ssDNA + EB, and **c** CQD2-ssDNA + EB + dsDNA

In Fig. 8, the 3D emission graphs of the developed sensor system are given for CQD2. The fluorescence emission of CQD2-ssDNA was observed at 465 nm and quenched by the addition of ethidium bromide. However, the CQD2 exhibited broad range emission in 3D plots. Then, the fluorescence emission intensity increased again both at 455 nm and 600 nm by the addition of complementary DNA.

**Fig. 9:**
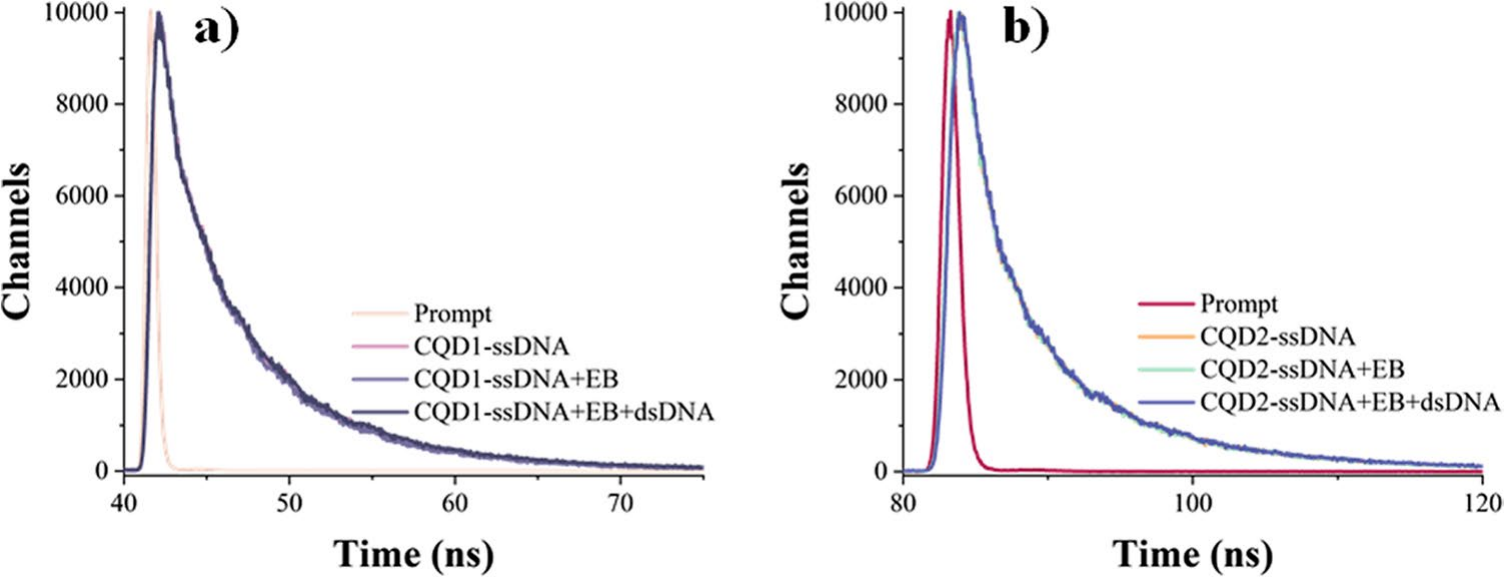
Fluorescence lifetime decays of carbon quantum dot–based biosensors in the presence of *H. pylori* genes (laser excitation source of 310 nm nanoled): **a** CQD1 and **b** CQD2

The fluorescence lifetime provides valuable information on the quenching mechanisms occurring in fluorescence sensors. The quenching mechanism occurs in two ways: dynamic (collisional) and static quenching mechanism. In dynamic quenching, the lifetime of the fluorophore decreases as quencher molecules interact with the excited fluorophore, leading to non-radiative decay. In static quenching, the quencher forms a non-fluorescent with the fluorophore in the ground state, typically not affecting the lifetime but reducing the overall fluorescence intensity. Additionally, the static quenching mechanism is independent of diffusion or molecular collisions [39]. The fluorescence lifetime graphs of the carbon quantum dot–based sensors developed for *H. pylori* genes are given in Fig. 9. The calculated lifetime values were found as 2.27 ns for CQD1 and 3.97 ns for CQD2. It was observed that the addition of ethidium bromide and complementary DNA to the system did not cause any changes in the lifetime durations. Based on these results, it has been determined that the mechanism of the developed sensor involves static quenching.

## Conclusions

Highly selective ratiometric fluorescence biosensors were successfully developed by using CQDs. The proposed biosensor system demonstrated the ability to distinguish between complementary and mismatched DNA sequences with high sensitivity and robust reproducibility. The novel biosensing system was developed using CQDs and ethidium bromide as a quencher for *H. pylori* associated genes detection. Comparative performance evaluations of the nanosensors revealed that CQD1 showed superior sensitivity compared to CQD2 for the detection. The quenching mechanism was determined to be primarily static quenching between CQDs and ethidium bromide molecules. The developed CQD1 and CQD2 sensor platforms exhibited better sensor behaviour compared to the other sensors presented in the literature. These findings highlight the potential of these nanosensor systems to drive innovation in optical biosensor technologies. The only limitation of the developed biosensor is that it requires the use of a DNA extraction kit, PCR process, and the conversion of double-stranded DNA to single-stranded DNA. Nevertheless, this method still demonstrates significant potential for integration into rapid-response and practical applications through the proposed biosensor strategy based on fluorometric techniques. In developed technology, this ratiometric biosensor system can be applied with smartphone and small UV light box, for detection *H. pylori* genes.

## Supplementary Information

Below is the link to the electronic supplementary material.

Supplementary file1 (DOCX 813 KB)

## Data Availability

No datasets were generated or analysed during the current study.
