# Supplementary material for: N-Doped carbon quantum dot–based ratiometric fluorescent nanosensor platforms for detection of gastric cancer-associated Helicobacter pylori genes
Source: Mikrochim Acta. 2025 Feb 12;192(3):147. doi: 10.1007/s00604-025-07004-4 (PMC11813823; doi:10.1007/s00604-025-07004-4)
Supplement: Supplementary file 1 — Supplementary file1 (DOCX 813 KB) [file 604_2025_7004_MOESM1_ESM.docx]

**Supporting Information**

**N- Doped Carbon Quantum Dots Based Ratiometric Fluorescent Nanosensor Platforms for Detection of Gastric Cancer-Associated *Helicobacter pylori* Genes**

Dilek Öztürk, Mahmut Durmuş^*^

*Gebze Technical University, Faculty of Science, Department of Chemistry, Gebze, 41400 Kocaeli, Türkiye*

Corresponding author E-mail: [durmus@gtu.edu.tr](mailto:durmus@gtu.edu.tr)

**Table of contents**

1. Synthesis of N-doped CQDs………………………………………………..…………………………….. S-2
2. Acid-Base Back Titration ……………………………………………………………………….….. ..........S-2
3. Table S1. Physical and Photophysical properties of the synthesized CQDs……………………………….S-2
4. Figure S1. Fluorescence signal changes of the CQD1 biosensor system in the presence of interfering materials (concentration of interfering materials: 0.1 M)…………………….………………….…………………………….……………………………......... ..S-3
5. Figure S2. Fluorescence signal changes of the CQD2 biosensor system in the presence of interfering materials (concentration of interfering materials: 0.1 M)……………………………………………....……………………………………………………..........S-4
6. Figure S3. The effect of interaction time between fluorescence intensity of biosesnsor system and concentration of cDNA and photostability study of a,c) CQD1, b,d) CQD2…………………………………………………………………..…………………….……….….…S-5
7. Table S2. Comparison of various studies of DNA detection biosensors…………………………..……….S-5
8. References…………………………………………………………………………………….……….……S-5

**Synthesis of N-doped CQDs**

N-doped CQDs synthesized hydrothermally in a microwave heater for 10 minutes at 350 Watt using the modified method applied by Ghirardello and his colleagues [1]. Citric acid and ethylenediamine, in a 1:1 molar ratio, used as precursors for the first synthesis, designated as CQD1, while malic acid and ethylenediamine, in the same molar ratio, used for the second synthesis, labelled as CQD2. The obtained brown crystals washed 4 times with a mixture of 5 mL methanol:acetone (1:1 v/v). The washed crystals dissolved in approximately 10 mL of water and placed on a low-molecular-weight cut-off (Mw=3.5 kDa) dialysis membrane. Purification performed at room temperature for 1 night and purified CQDs recrystallized.

**Acid-Base Back Titration**

The acid-base titration method, as described by Zhang et al., was employed to determine the amount of -COOH groups on the surface of the synthesized N-doped CQDs based on back titration [2]. A 2 mL solution containing the synthesized N-doped CQDs was prepared with a concentration of 1000 ppm. Then, 2 mL of 0.01 M NaOH solution was added to this solution and stirred for 2 hours at room temperature. During this process, all the carboxylic acid groups present were converted to sodium salts (reaction (1)), establishing an equilibrium between the N-doped CQDs and NaOH once the pH stabilized. The excess NaOH in the solution was then titrated with a 0.01 M HCl solution using phenolphthalein as an indicator (reaction (2)). After measuring the amount of HCl solution consumed, the amount of NaOH solution that reacted with the -COOH groups on the surface of the N-doped CQDs in the first reaction was calculated. Each titration was performed in at least two replicates.

-COOH + NaOH → -COONa + H_2_O reaction (1)

HCl + NaOH → NaCl + H_2_O reaction (2)

**Table S1.** Physical and Photophysical properties of the synthesized CQDs

| **CQDs** | **μmol -COOH /mg CQDs** | **%Q_Fy_** | **Abs (nm)** | **Ex (nm)** | **Em (nm)** | **Zeta Potential (mV)** | **pH** |
| --- | --- | --- | --- | --- | --- | --- | --- |
| **CQD1** | 2.025 ±0.075 | 23.8 | 240, 350 | 360 | 450 | -4.01 | 4.32 |
| **CQD2** | 0.885± 0.05 | 15.3 | 340, 450 | 360 | 460 | 0.371 | 5.93 |


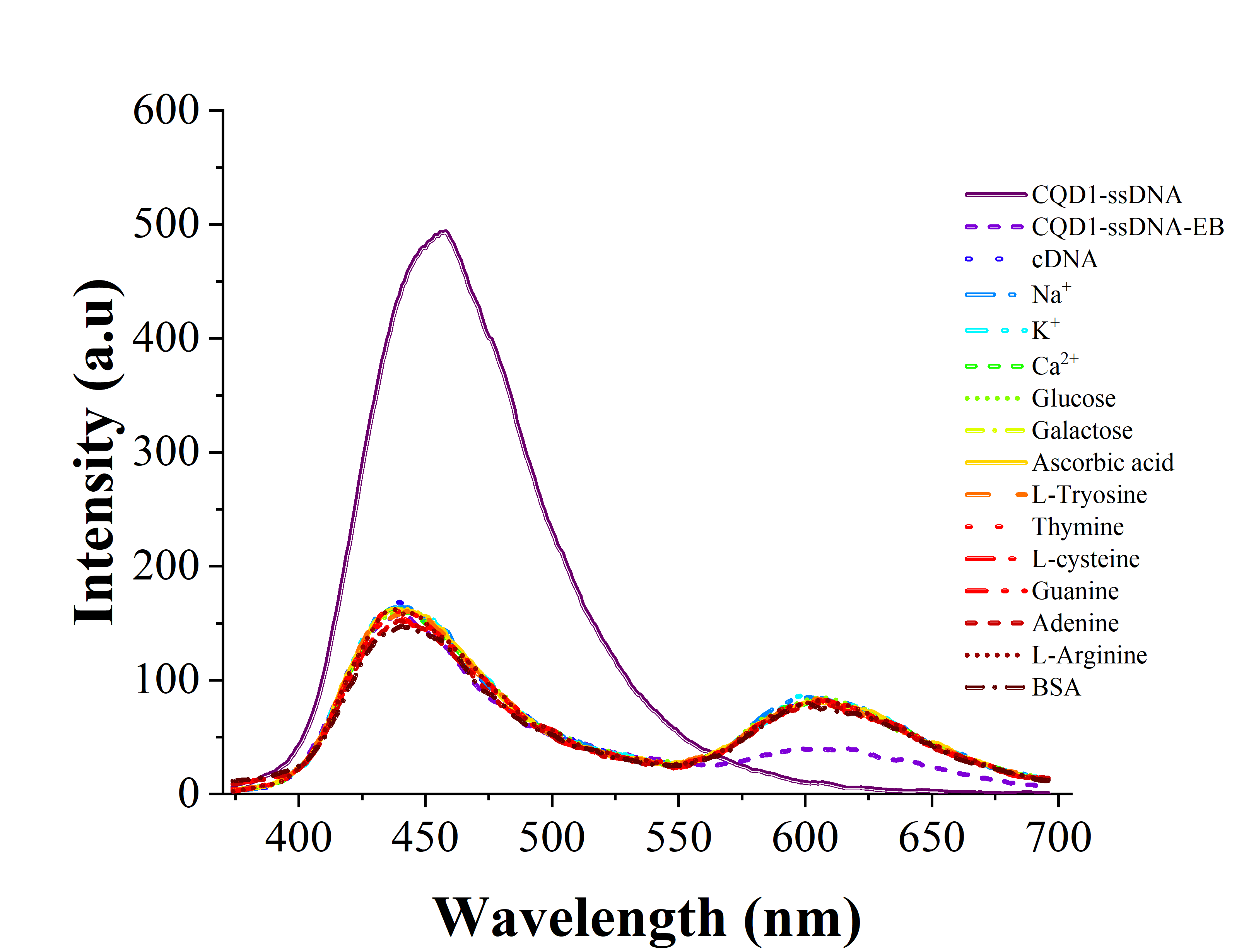


Figure S1. Fluorescence signal changes of the CQD1 biosensor system in the presence of interfering materials (concentration of interfering materials: 0.1 M).


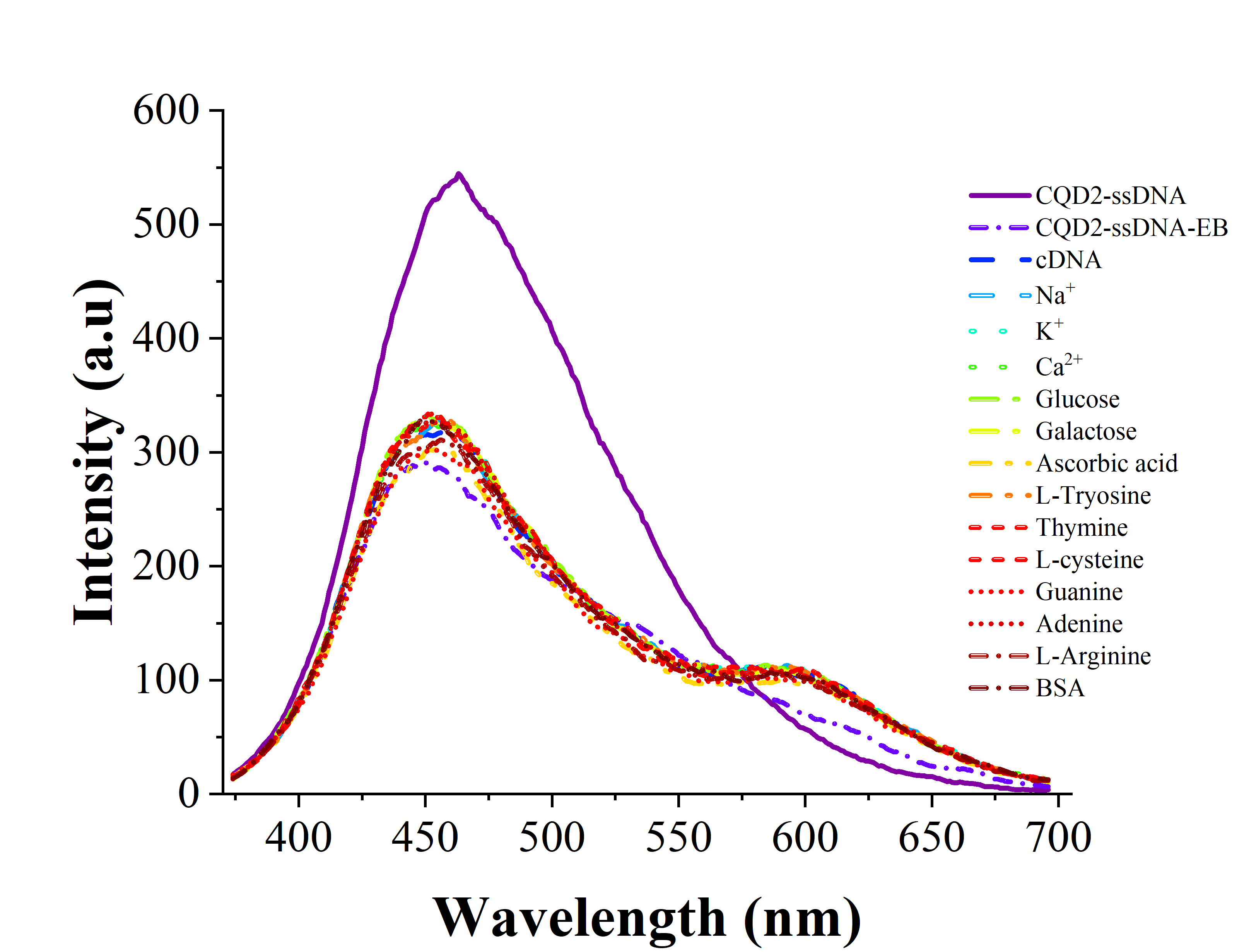


Figure S2. Fluorescence signal changes of the CQD2 biosensor system in the presence of interfering materials (concentration of interfering materials: 0.1 M).


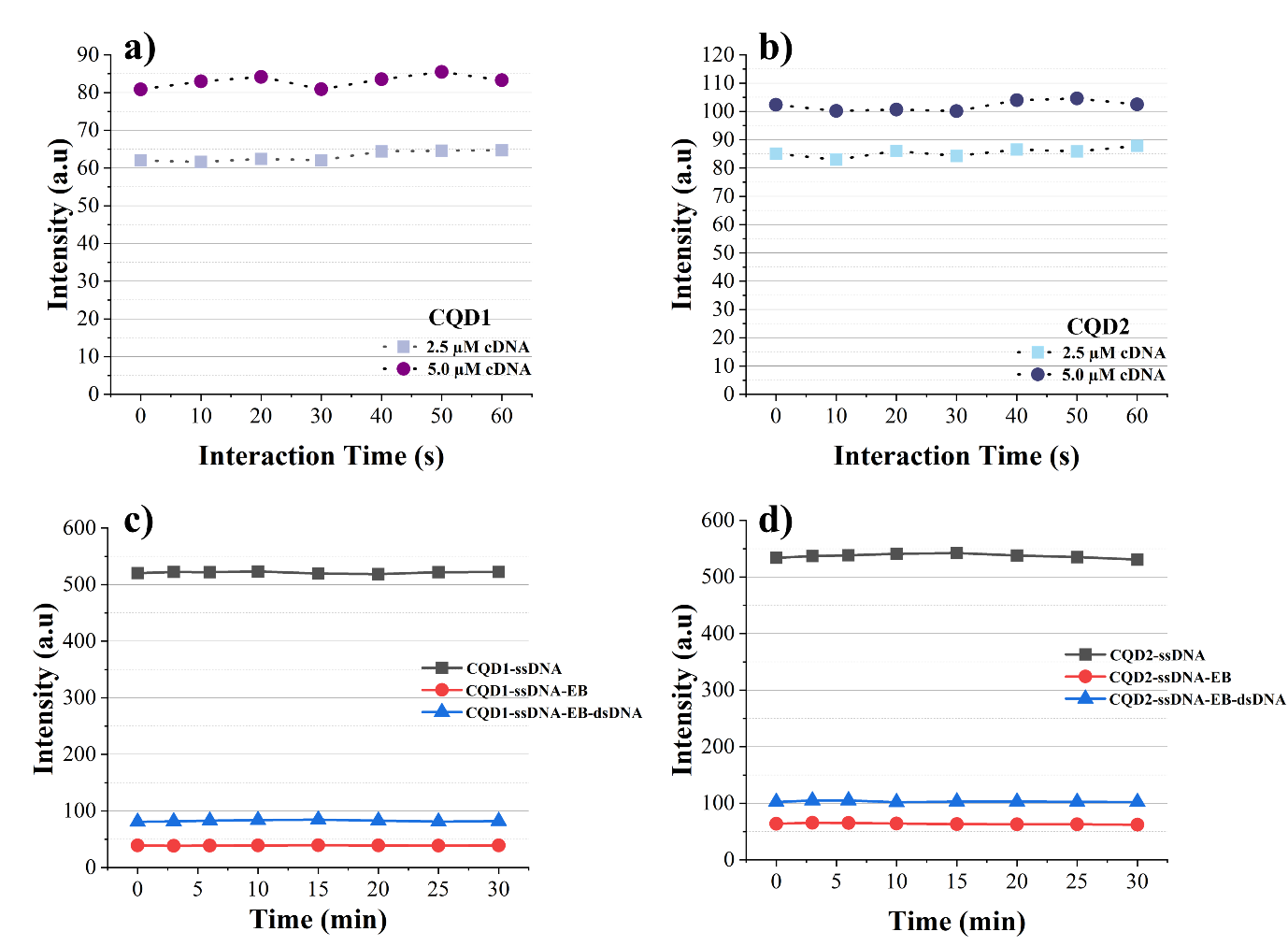


Figure S3. The effect of interaction time between fluorescence intensity of biosesnsor system and concentration of cDNA and photostability study of a,c) CQD1, b,d) CQD2

**Table S2.** Comparison of various studies of DNA detection biosensors

| **Materials** | **Method** | **LOD** | **References** |
| --- | --- | --- | --- |
| Carbon dots | Optical | 0.47 μM (not selective) | [3] |
| Carbon dots | Optical | 1 μM (not selective) | [4] |
| Silver nanoparticles | Optical | 0.1 μg (not selective) | [5] |
| Silicon nanodots | Optical | 4.3 nM (not selective) | [6] |
| N-doped CQDs | Optical | 0.098 μM (Selective for H.pylori genes) | This work |

**References**

(1) Ghirardello M, Shyam R, Liu X, Garcia-Millan T, Sittel I, Ramos-Soriano J, Kurian KM ,Galan MC (2022) Carbon dot-based fluorescent antibody nanoprobes as brain tumour glioblastoma diagnostics. Nanoscale Adv 4(7):1770-1778. [https://doi.org/10.1039/d2na00060a](https://doi.org/10.1039/D2NA00060A)

(2) Zhang Y, Song J, Yang S, Ouyang J, Zhang J (2022) Carbon Nanostructure-Based DNA Sensor Used for Quickly Detecting Breast Cancer-Associated Genes. Nanoscale Res Lett 17 (1):93. <https://doi.org/10.1186/s11671-022-03730-3>

(3) Huang S, Wang L, Zhu F, Su W, Sheng J, Huang C, Xiao Q (2015) A ratiometric nanosensor based on fluorescent carbon dots for label-free and highly selective recognition of DNA. RSC Adv 5(55):44587-44597. [https://doi.org/10.1039/c5ra05519a](https://doi.org/10.1039/C5RA05519A)

(4) Bai W, Zheng H, Long Y, Mao X, Gao M, Zhang L (2011) A carbon dots-based fluorescence turn-on method for DNA determination. Analytical sciences : the international journal of the JSAC 27(3):243-246. <https://doi.org/10.2116/analsci.27.243>

(5) Dragan AI, Bishop ES, Strouse RJ, Casas-Finet JR, Schenerman MA, Geddes CD (2009) Metal-enhanced ethidium bromide emission: Application to dsDNA detection. Chem Phys Let, 480(4-6):296-299. <https://doi.org/10.1016/j.cplett.2009.09.005>

(6) Zhang Y, Hou D, Zhao B, Li C, Wang X, Xu L, Long T (2020) Ratiometric fluorescence detection of DNA based on the inner filter effect of Ru (bpy) 2 (dppx) 2+ toward silicon nanodots. ACS Omega, 6(1):857-862. <https://doi.org/10.1021/acsomega.0c05434>
